# Gut Microbiota Diversity in 16 Stingless Bee Species (Hymenoptera: Apidae: Meliponini)

**DOI:** 10.3390/microorganisms13071645

**Published:** 2025-07-11

**Authors:** María de Lourdes Ramírez-Ahuja, Kenzy I. Peña-Carrillo, Mayra A. Gómez-Govea, Mariana Lizbeth Jiménez-Martínez, Gerardo de Jesús Trujillo-Rodríguez, Marisol Espinoza-Ruiz, Antonio Guzmán Velasco, Adriana E. Flores, José Ignacio González-Rojas, Diana Reséndez-Pérez, Iram Pablo Rodríguez-Sánchez

**Affiliations:** 1 Laboratorio de Fisiología Molecular y Estructural, Facultad de Ciencias Biológicas, Universidad Autónoma de Nuevo León, San Nicolás de los Garza 64460, Mexico; lulu.ahuja@hotmail.com (M.d.L.R.-A.); mayragee@gmail.com (M.A.G.-G.); mariana.jimenez80@gmail.com (M.L.J.-M.); entogerry36@gmail.com (G.d.J.T.-R.); 2 Campo Experimental General Terán, Instituto Nacional de Investigaciones Forestales Agrícolas y Pecuarias, Km 31 Carretera Montemorelos-China, General Terán 67400, Mexico; kenzy.p@gmail.com; 3 Facultad de Ciencias Químicas, Campus IV, Universidad Autónoma de Chiapas, Tapachula 30580, Mexico; marisol.espinoza@unach.mx; 4 Laboratorio de Conservación de Vida Silvestre y Desarrollo Sustentable, Facultad de Ciencias Biológicas, Universidad Autónoma de Nuevo León, San Nicolás de los Garza 64460, Mexico; antonio.guzman@uanl.mx (A.G.V.); jose.gonzalezr@uanl.mx (J.I.G.-R.); 5 Laboratorio de Entomología Médica, Departamento de Zoología de Invertebrados, Facultad de Ciencias Biológicas, Universidad Autónoma de Nuevo León, San Nicolás de los Garza 66455, Mexico; adriana.floressr@uanl.edu.mx; 6 Departamento de Biología Celular y Genética, Facultad de Ciencias Biológicas, Universidad Autónoma de Nuevo León, San Nicolás de los Garza 66455, Mexico

**Keywords:** stingless bees, corbiculate bees, microbiome, gut microbiota

## Abstract

Bacterial symbionts play an important role in insect survival by contributing to key metabolic and defensive functions. While stingless bees are known to harbor diverse microbial communities, their core bacterial symbionts remain poorly characterized. In this study, we analyzed the gut microbiota of sixteen stingless bee species collected from different regions of Mexico using 16S rRNA gene sequencing on the Illumina^®^ MiSeq™ platform. Our results revealed that Proteobacteria, Firmicutes, and Actinobacteria are the most abundant bacterial phyla across species. Among the dominant genera, lactic acid bacteria, such as *Lactobacillus* spp., *Bifidobacterium*, and *Fructobacillus* spp., were the most prevalent. These bacteria are responsible for developing biochemical functions in metabolic processes like lactic fermentation and the biotransformation of complex organic compounds into molecules that are more easily assimilated by bees. This study offers a novel perspective on the diversity and predicted composition of gut microbiota in Mexican stingless bees. By highlighting differences in microbial communities among species with different feeding habits, our results emphasize the importance of preserving microbial biodiversity in these pollinators.

## 1. Introduction

The tribe Meliponini (Hymenoptera: Apidae), commonly known as stingless bees, comprises over 60 genera and hundreds of species, including *Melipona*, *Trigona*, *Tetragonula*, and *Scaptotrigona* and is distributed across tropical and subtropical regions worldwide [1,2]. In the Neotropics, stingless bees are key pollinators that contribute to ecosystem resilience and biodiversity maintenance [1]. Their evolutionary history dates back approximately 80 million years to the Cretaceous period; thus, a long trajectory has shaped their remarkable taxonomic and ecological diversification [2]. Phylogenetic reconstructions have revealed how historical biogeographic and climatic changes have driven lineage diversification within Meliponini [2,3].

The ecological and agricultural relevance of stingless bees extends beyond pollination. Their social organization enhances foraging efficiency, especially for crops like coffee and cacao, while their honey is culturally significant and has been used for its therapeutic properties, including antibacterial and anti-inflammatory effects [4]. However, despite their importance, stingless bees face significant threats from habitat degradation, agrochemical exposure, emerging pathogens, and interspecific competition, especially with the invasive *Apis mellifera*, which affects native pollinator populations by competing for nesting and floral resources [5,6,7,8,9,10].

While numerous studies have characterized the gut microbiota of the Western honeybee, *A. mellifera*, uncovering a consistent core composed of *Spodgrassella alvi*, *Gillamella apicola*, *Frischella perrara*, *Bifidobacterium*, and clades of *Lactobacillus* (Firm-4 and Firm-5), as well as other lesser-known symbionts like *Apibacter* and *Parasaccaribacter* [11,12,13,14,15], the gut microbial communities of stingless bees remain poorly characterized. Notably, *Snodgrassella* and *Gilliamella*, which are tightly associated with corbiculate bee linage, appear to be absent or only sporadically detected in stingless bees [11,13,14].

Recent studies have begun to address this gap. Investigations on Australian stingless bees revealed that although lactic acid bacteria (LAB) are commonly found across genera, such as *Teragonula* and *Austroplebeia*, the composition of LAB is shaped by phylogenetic lineage and environmental context, suggesting both host-specific and regionally influenced patterns [16]. LAB isolated from Mexican stingless bees, including *Melipona beecheii*, *Scaptotrigona pectoralis*, and *Plebeia jatiformis*, have been shown to include members of *Apilactobacillus*, *Lactiplantibacillus*, *Weissella*, and *Leuconostoc*, highlighting a diverse and functionally relevant symbiotic assemblage [17].

Given the ecological relevance of stingless bees, their diversity in Mexico, and the limited data available on their microbial symbionts, this study aims to characterize the gut microbiota of 16 stingless bees using high throughput 16S rRNA sequencing. Readers must be aware that the scope of our inferences is mainly limited by an unreplicated design. Notwithstanding, this is, to our knowledge, the first exploration study in Mexico to profile the microbiota of Meliponini using next-generation sequencing (NGS), providing a baseline for future research on microbial ecology, conservation, and bee health.

## 2. Materials and Methods

### 2.1. Stingless Bee Collection and Identification

**Figure 1:**
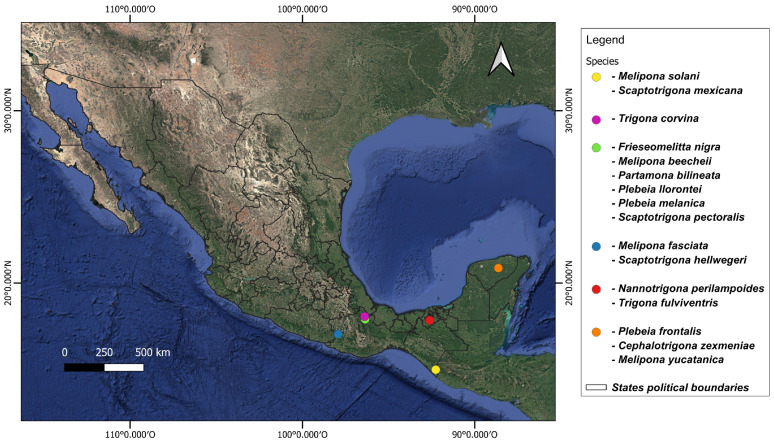
Sampling sites of stingless bees collected from southern Mexico.

Adult worker bees were obtained from different states in the south of Mexico between May 2021 and October 2023 (Figure 1, Table S1). Specimens were identified based on morphological characteristics with the keys of Ayala-Barajas [18,19] and Arnold et al. [20]. Ten worker individuals per species were sampled directly from hives and stored in a DNA shield until processing.

### 2.2. Gut Collection

The guts from ten worker stingless bees per species were used for tissue isolation. Prior to dissection, adult bees were surface-sterilized with 96% ethanol for three minutes and then rinsed with sterile deionized water. Dissections were performed under a stereomicroscope (Leica MZ16, 1.6X, Wetzlar, Germany) on sterile Petri dishes containing 2 mL of sterile phosphate-buffered solution (10 mM, pH 7.4, Ambion, Thermo Fisher Scientific, Waltham, MA, USA), using a pair of flamed-sterilized needles cooled in 96% ethanol. The dissected guts were transferred into 1.5 mL Eppendorf^®^ tubes containing a DNA shield and stored at 70 °C until DNA extraction and sequencing.

### 2.3. DNA Extraction and Sequencing

The gut samples were processed using the ZymoBIOMICS^®^ service provided by Zymo Research (Irvine, CA, USA). DNA was extracted using the ZymoBIOMICS^®^-96 MagBead DNA Kit (Zymo Research). Library preparation followed the protocol described by Gómez-Govea et al. [21], and sequencing was performed on an Illumina^®^ MiSeq™ platform as part of the ZymoBIOMICS™ Service Targeted Metagenomic Sequencing service. Bacterial 16S rRNA gene sequencing targeted the V3–V4 region using the Quick-16S™ NGS Library Prep Kit (Zymo Research).

### 2.4. Bioinformatic Analysis

The processing of the 16S rRNA gene libraries was carried out using the DADA2 pipeline. Reads were filtered based on quality, and singletons, chimeric sequences, and low-quality reads were removed. Amplicon Sequence Variants (ASVs) were inferred using default parameters [22]. Metadata was then imported to create a phyloseq object (v1.46.0) for downstream analysis in R [23]. ASVs were filtered based on a prevalence threshold of 0.05, retaining only those with a relative abundance greater than 5%. Samples were rarefied to 10,000 reads, and the read counts were transformed into relative abundances. Alpha diversity (Shannon and Chao indexes) and beta diversity (Principal Coordinate Analysis, PCoA) were performed using the microbiome v1.22.0, vegan v2.6-4, stats v4.3.2, and ggplot2 v3.4.4 packages in Rstudio v12.1. Statistical differences in alpha diversity were assessed using a standard *t*-test (stats v4.3.2). Pairwise comparisons were conducted only among bee genera represented by more than one species: *Melipona*, *Plebeia*, *Trigona*, and *Scaptotrigona*.

The top 20 bacterial taxa at the phylum, family, genus, and species levels were visualized using stacked bar plots. PCoA based on Bray–Curtis distances was performed to visualize differences between groups in beta diversity. Additionally, differences in taxa abundance between the genera *Melipona* and *Trigona* were explored through marker bacteria analysis, motivated by their distinct feeding behaviors. Differences between *M. beecheii* (an obligate pollinivore) and *T. corvina* (a facultative necrophage) were tested. Differentially abundant ASVs were identified using DEseq2, fold changes (FC > 1), and the False Discovery Rate (FDR) > −log (0.05) was visualized via volcano plots generated with EnhancedVolcano v1.18.0 [24,25].

## 3. Results

**Figure 2:**
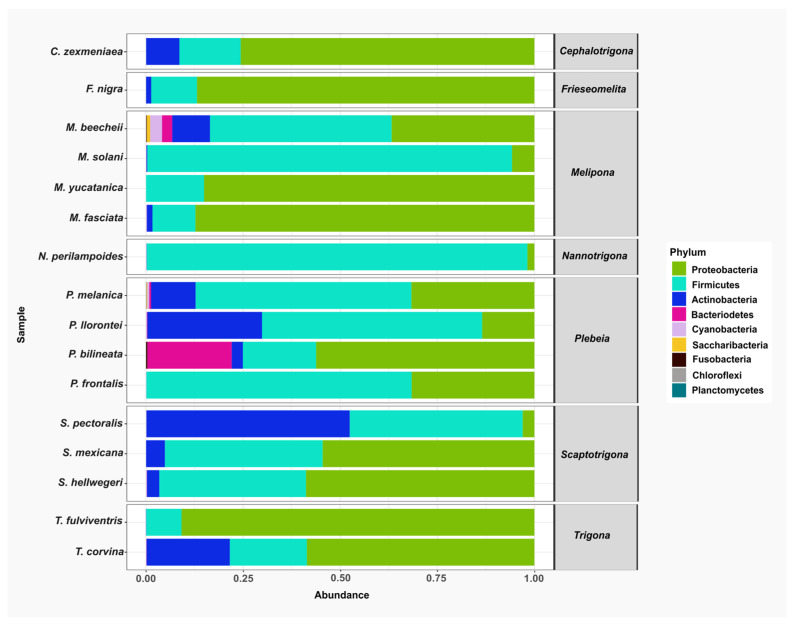
The relative abundance of bacterial phyla in gut samples from stingless bee species. Bar plots represent the composition of the most abundant phyla across the bee species analyzed. The predominant phyla were Proteobacteria (green), Firmicutes (aqua), and Actinobacteria (blue).

A total of nine bacterial phyla were identified across all stingless bee species: Proteobacteria, Firmicutes, Actinobacteria, Bacteroidetes, Cyanobacteria, Saccaribacteria, Fusobacteria, Chloroflexi. and Planctomycetes. Among these, Proteobacteria, Firmicutes, and Actinobacteria were the most abundant and were detected in nearly all the species analyzed. In contrast, Chloroflexi, Planctomycetes, Saccharibacteria, and Fusobacteria were found in only three species and represented the least abundant phyla (Figure 2).

At the class level, Bacilli was among the top 20 most abundant classes and was consistently detected in all the species analyzed. Other frequently observed classes included Actinobacteria, which were present in *Scaptotrigona pectoralis*, *S. mexicana*, *S. hellwegeri*, *Plebeia melanica*, *P. llorontei*, *P. bilineata*, *Melipona beechii*, *M. solani*, *M. fasciata*, *Trigona corvina*, *Frieseomelitta nigra*, and *Cephalotrigona zexmeniae*. Within the Proteobacteria group, Alphaproteobacteria was found in all stingless bee species analyzed.

**Figure 3:**
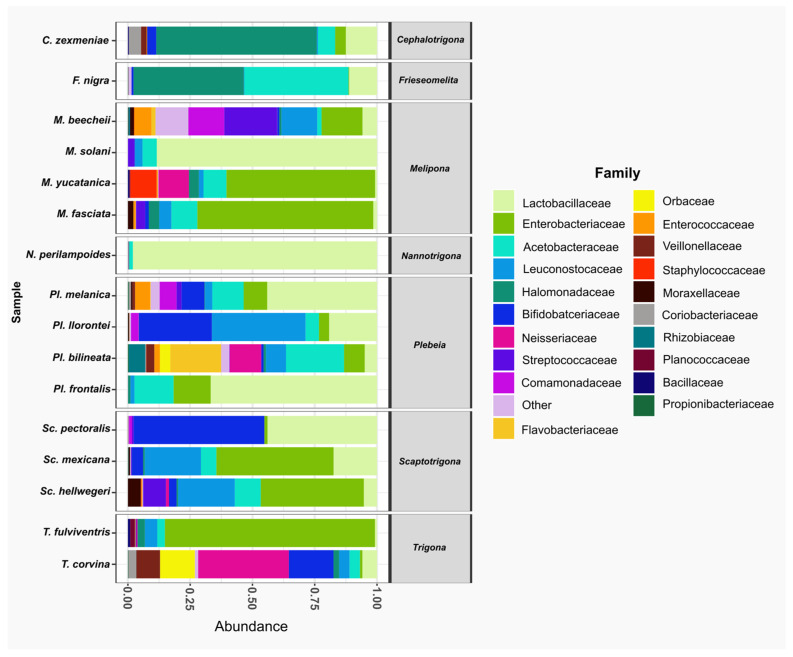
The relative abundance of bacterial families in gut samples from stingless species. Bar plots represent the composition of the most abundant families across the bee species analyzed. The predominant family was Lactobacillaceae (light green), present in all species. Other frequent families included Enterobacteriaceae (green) and Acetobacteraceae (aqua).

At the family level, Lactobacillaceae was the most abundant family, present in all species. It was the dominant family in *Nannotrigona perilampoides* and *Melipona solani*, while in *M. yucatanica* and *Trigona fulviventris*, it was the least abundant. The second most abundant family was Enterobacteriaceae, also present in all bee species, followed by Acetobacteraceae, which was absent only in *Scaptotrigona pectoralis*. In general, within the top 20 families, *T. corvina*, *P. bilineata*, and *M. beecheii* had microbiomes composed of 18, 17, and 16 families, respectively (Figure 3).

From the aforementioned families, the 20 most abundant genera were *Lactobacillus*, *Bifidobacterium*, *Weissella*, *Enterobacter*, *Carnimonas*, *Serratia*, *Pantoea*, *Saccharibacter*, *Snodgrassella*, *Bombella*, *Fructobacillus*, *Providencia*, *Zymobacter*, *Rosenbergiella*, *Commensalibacter*, *Gilliamella*, *Cronobacter*, *Pectinatus*, *Alysiella*, and *Acetobacter*.

**Figure 4:**
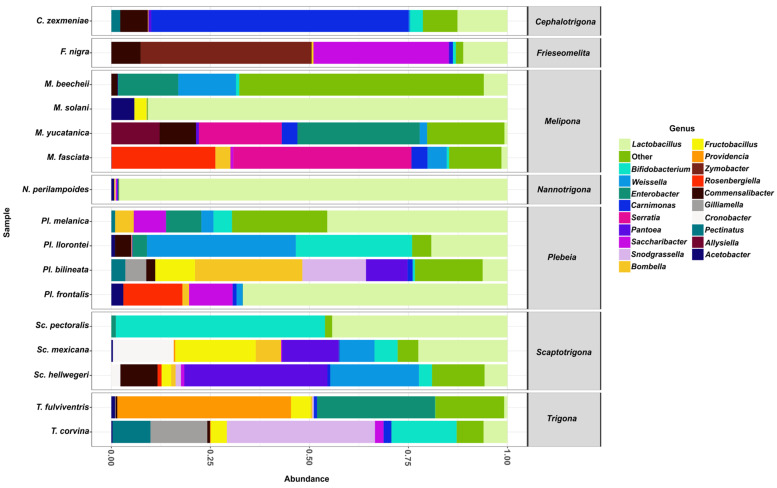
The relative abundance of bacterial genera in gut samples from stingless bee species. Bar plots represent the composition of the most abundant bacterial genera across all bee species analyzed. *Lactobacillus* (light green) was the dominant genus in most species.

Among these genera, *Lactobacillus* was found in all stingless bee species analyzed (Figure 4) and was represented by five distinct species: *Lactobacillus* sp29216, *Lactobacillus suebicus*, *Lactobacillus* sp29206, *Lactobacillus oris*, and *Lactobacillus vaccinostercus*. L. sp29216 was present in all 16 bee species, whereas *L. vaccinostercus* was found only in *P. llorontei*, *M. solani*, *N. perilampoides*, *F. nigra*, *P. frontalis*, and *M. fasciata*.

The second most abundant genus was *Bifidobacterium*, which was detected in most species except for *N. perilampoides*, *M. yucatanica*, and *P. frontalis*. This genus was represented by a single species, *Bifidobacterium* sp4969, which was found to be present in most species except for *N. perilampoides*, *M. yucatanica*, and *P. frontalis*. The third most abundant genus was *Weissella*, present in most species except *S. pectoralis*, *T. fulviventris*, *M. solani*, *N. perilampoides*, and *F. nigra*. It was represented by the species *Weissella paramesenteroides*, found in several species, including *P. melanica*, *M. beecheii*, *P. llorontei*, *S. mexicana*, *P. bilineata*, *M. yucatanica*, *C. zexmeniae*, *P. frontalis*, *M. fasciata*, and *S. hellwegeri*.

**Figure 5:**
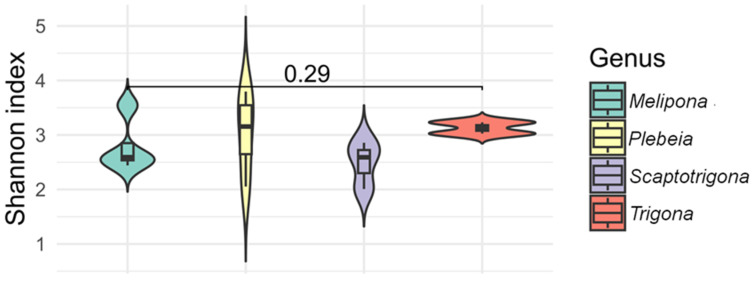
The alpha diversity (Shannon index) of gut bacterial communities across stingless bee genera. Violin plots show the distribution of Shannon index values for species within the genera *Melipona*, *Plebeia*, *Scaptotrigona*, and *Trigona*. All four genera exhibited relatively high bacterial diversity, with no statistical differences observed between them (*p* = 0.29).

The alpha diversity index (Shannon) indicated that species from the genera *Melipona*, *Plebeia*, *Scaptotrigona*, and *Trigona* exhibited the most diverse bacterial communities, with values ranging from 1.9 to 3.7. Within *Melipona*, *M. beecheii* exhibited the highest diversity, while *M. solani*, *M. yucatanica*, and *M. fasciata* showed similar values. In *Plebeia*, *P. melanica* and *P. bilineata* had similar values (3.4 and 3.7, respectively), as did P. *llorontei* and *P. frontalis* (2.0 and 2.8, respectively). Alpha diversity values in *Scaptotrigona* species were approximately 2.0, and values in *Trigona* species were around 3.0 (Figure 5). No statistically significant differences were observed for alpha diversity (*p* > 0.05). The species with the highest observed bacterial richness were *Partamona bilineata*, followed by *M. beecheii*, *Plebeia melanica*, *Trigona fulviventris*, and *T. corvina*, whereas *Scaptotrigona pectoralis* and *Frieseomelitta nigra* exhibited the lowest diversity.

The Chao index revealed that *P. bilineata* had the highest estimated bacterial richness, followed by *T. corvina* and *M. beecheii*. In contrast, *S. pectoralis* and *P. frontalis* exhibited the lowest richness, with 39 and 53 observed species, respectively.

**Figure 6:**
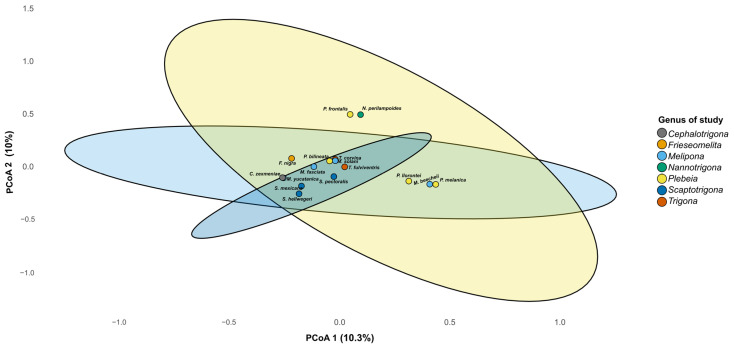
Principal coordinate analysis (PCoA) based on Bray–Curtis distances of gut microbiota from stingless bee genera. The PCoA plot shows the partial clustering of bacterial community composition according to bee genus. Each point represents the microbiome of one species, and ellipses indicate the 95% confidence interval for each genus. The first two axes explain 20.3% of the total variance (PCo1 = 10.3%, PCo2 = 10.0%).

Beta diversity analysis revealed differences in microbiome composition among bee genera, with three clustering patterns observed on PCoA 1 in the plot (Figure 6). One cluster was composed of *P. llorontei*, *P. melanica*, and *M. beecheii*; a second cluster was composed of *P. frontalis* and *N. perilampoides*; and a third cluster included the rest of the taxa. These differences were statistically supported by the PERMANOVA (R^2^ = 0.43, F = 1.13, *p* = 0.009). On PCoA 2, *P. frontalis* and *N. perilampoides* were grouped and separated from the rest of the taxa.

### Bacteria Marker Analysis of Bacterial Communities in Melipona and Trigona

A comparative analysis of the bacterial communities of *Melipona* and *Trigona* species was carried out to identify marker bacteria, considering differences in their foraging behavior. For example, *M. beecheii* forages exclusively on plants and is managed for honey production, whereas *T. corvina* is a facultative species that also feed on carrion. *Melipona* and *Trigona* share several bacterial genera, including *Lactobacillus* spp., *Citrobacter* spp., *Enterobacter* spp., and *Klebsiella* spp. However, species-level comparisons revealed distinct patterns of abundance.

In *Melipona*, the most abundant bacterial species included *Acinetobacter apisnectaris*, *Actinobacillus capsulatus*, *Aquabacterium parvum*, *Asaia astilbis*, *Bergeyella zoohelcum*, *Citrobacter rodentium*, *Delftia lacustris-tsuruhatensis*, *Enterobacter ludwigii*, *Fluviicola* sp16240, *Lactobacillus* sp29166, *Lactobacillus* sp29206, *Lactobacillus* sp29216, *Lactobacillus suebicus*, *Lactobacillus vaccinostercus*, *Moraxella cuniculi*, *Pedobacter insulae*, *Prevotella timonensis*, *Pseudomonas otitidis*, *Ralstonia pickettii*, *Roseburia* sp33131, *Serratia marcescens* and *Weissella paramesenteroides*. These taxa were less abundant in *Trigona*.

In contrast, the most abundant bacterial species in Trigona included *Bifidobacterium callitrichos*, *Bifidobacterium* sp4969, *Bifidobacterium* sp4969, *Citrobacter amalonaticus*, *Enterobacter aerogenes*, *Escherichia coli*, *Fructobacillus ficulneus*, *Fructobacillus pseudoficulneus*, *Gilliamella apicola*, *Gluconobacter oxydans-uchimurae*, *Klebsiella pneumonia*, *Lactobacillus mellis*, *Lactobacillus* sp29206, *Pectinatus frisingensis*, *Proteus mirabilis*, *Providencia rettgeri-vermicola*, *Saccharibacter* sp45729 and *Snodgrassella alvi*.

## 4. Discussion

In this study, we report for the first time the characterization of the gut microbiota of sixteen Mexican stingless bees (Apidae: Meliponini). Despite the fact that differences in alpha diversity were not found, beta diversity suggested that microbiome composition differed significantly for some taxa. For example, the grouping of *P. llorontei*, *P. melanica*, and *M. beecheii* may indicate differences linked to diet, as they were reared by the same beekeeper. On the other hand, *P. frontalis* and *N. perilampoides* were collected at different locations on the side of the Gulf of Mexico. Moreover, the exploratory analysis contrasting feeding habits suggests differences in microbiome composition. Because our hypothesis is limited by the lack of replicates, which may, for instance, lead to hidden natural biological variation, a higher risk of statistical errors, or weak conclusions [26], our exploratory results must be used with caution. Despite this, they serve as a reference for further studies on the microbiome of Mexican stingless bees.

The gut microbiota plays essential roles in digestion, the synthesis of bioactive compounds, and immune protection and is key to individual well-being and colony cohesion [12]. However, anthropogenic factors such as habitat fragmentation, pollution, and pesticides have been shown to significantly alter the composition and functionality of this microbiota in social bees, affecting their ability to metabolize nutrients, resist infections, and adapt to environmental changes [27]. We found that Firmicutes, Actinobacteria, and Proteobacteria were the most dominant and conserved phyla across all species analyzed.

Within the order Hymenoptera, most studies on gut bacterial communities have focused on *Apis mellifera*, where five core species of bacteria have been consistently identified in the intestinal tract: *Snodgrassella alvi*, *Gilliamella apicola*, *Lactobacillus* spp., and *Bifidobacterium* spp. These bacteria are commonly found in the adult worker *A. mellifera* worldwide [27].

It has been suggested that the emergence of eusocial corbiculate bees (a group of bees that have pollen baskets on their hind legs) coincided with the acquisition of the following key gut bacterial lineages: *Snodgrassella*, *Gilliamella*, *Lactobacillus* (Firm-4 and Firm-5), and *Bifidobacterium*. However, our findings indicate that not all stingless bee species harbor these genera. For example, *Snodgrasella* and *Gilliamella* were found in *Trigona corvina* and *Partamona bilineata* but not in *Melipona* species. This observation is consistent with previous reports describing the absence of *Snodgrassella* and *Gilliamella* in members of the Meliponini tribe [15,28,29,30].

Recently, Cerqueira et al. [15] demonstrated that the gut microbiota of *Partamona helleri*, *Plebeia droyana*, *Trigona spinipes*, *Tetragonula angustula*, and *T. carbonaria* included both *Snodgrassella* and *Gilliamella*, whereas *Melipona* spp. lacked these bacteria, consistent with our observations. Hall et al. [31] suggest that these genera have been replaced in *Melipona* spp. by new symbionts that fulfill equivalent or even enhanced metabolic functions. However, a study by Sarton-Lohéach et al. [32] reported the presence of *Snodgrasella* in some *Melipona* colonies. This suggests that the genus is not entirely absent in *Melipona* but may be occasionally acquired from other bees, with its prevalence potentially varying according to season, bee age, and colony development. The microbiota of Meliponini is highly variable, which may reflect the evolutionary diversity of their life histories, morphologies, and behaviors. No single bacterial phylotype has been consistently reported across all bee species [33].

One of the earliest microbiome studies on the intestinal tract of stingless bees was conducted on *Melipona quadrifasciata*, where *Bacillus meliponotrophicus* was found to be associated with *Trigona* and *Melipona* but not with *Apis* or *Bombus*, despite their phylogenetic proximity to stingless bees [32]. Later, Nogueira-Neto [34] suggested that *B. meliponotrophicus* was likely involved in the pre-digestion of honey and pollen in *M. quadrifasciata*. Additional studies also identified *Bacillus* species in *M. quadrifasciata* [35,36,37].

Among the few studies conducted in Mexico on the effects of lactic acid bacteria (LAB), Torres-Moreno et al. [17] found that LAB can provide beneficial effects, including reducing bacterial and parasitic infections and increasing honey production in hives. They analyzed the gut microbiota of *Melipona beecheii*, *Scaptotrigona pectoralis*, *Plebeia llorentei*, and *Plebeia jatiformis*, identifying the presence of *Lactiplantibacillus plantarum*, *Weissella paramesenteroides*, *Leuconostoc citreum*, and *Apilactobacillus*. These authors demonstrated that *L. plantarum*, *W. paramesenteroides*, and *L. citreum* are heterofermentative and exhibit a strong capacity to hydrolyze complex plant-derived carbohydrates present in pollen and other plant structures, such as arabinose, cellobiose, fructose, maltose, mannitol, melibiose, raffinose, sucrose, sorbitol, and xylose [17,38,39]. This metabolic capacity has been associated with the feeding habits of bees, which provide bacteria with a nutrient-reach environment derived from pollen and nectar [40]. The ability to ferment a wide variety of carbohydrates is considered a desirable trait for potential zootechnical probiotic applications in bees, as LAB can enhance the assimilation of nutrients from plant-based sources [38,40].

Torres-Moreno et al. [17] determined that these bees possess a resilient microbiota related to their specific environmental conditions and diet. Furthermore, the isolated bacterial strains may contribute to honey production or fermentation, making them potentially valuable for fermented food processing or as zootechnical probiotics for bees. Among the bacteria reported by these authors, *W. paramesenteroides* was also identified in our study in several species, including *Plebeia* spp., *Scaptotrigona hellwegeri*, *S. pectoralis*, *Melipona beecheii*, and *M. yucatanica*. It is possible that this bacterium performs similar functions in these species as previously described in other stingless bees. Another species found in our study was *Bombella intestine*, which was found in *Partamona bilineata*, *Plebeia melanica*, and *Scaptotrigona mexicana*. *B. intestini* has been reported to produce acids from various carbohydrates, including sucrose, d-fructose, d-glucose, d-mannitol, d-galactose, d-mannose, and l-arabinose [41].

Several studies suggest that lactic acid bacteria, such as *Lactobacillus* spp., *Bifidobacterium*, and *Fructobacillus* spp., are among the most abundant groups in the hindgut of stingless bees. These bacteria are responsible for developing biochemical functions in metabolic processes, including lactic fermentation and the biotransformation of complex organic compounds into molecules that are more easily assimilated by bees. In addition, they act as probiotics, helping to prevent digestive infections [16,27,28,30,42,43,44,45]. In our study, *Lactobacillus* was present in all species analyzed; *Bifidobacterium* was the second most abundant genus; and *Weissella* ranked third. Other studies on the microbiome of stingless bees have been conducted in species such as *Lepidotrigona terminata*, *Lepidotrigona ventralis*, and *Tetragonula pagdeni*, where bacteria including *Acetobacter*, *Snodgrassella*, *Lactobacillus*, *Psychrobacter*, *Pseudomonas*, and *Bifidobacterium* have been identified. The relative abundance of these bacterial groups varied among communities within the same bee species, likely influenced by multiple factors such as dietary preferences, age, and genetic differences [15].

**Figure 7:**
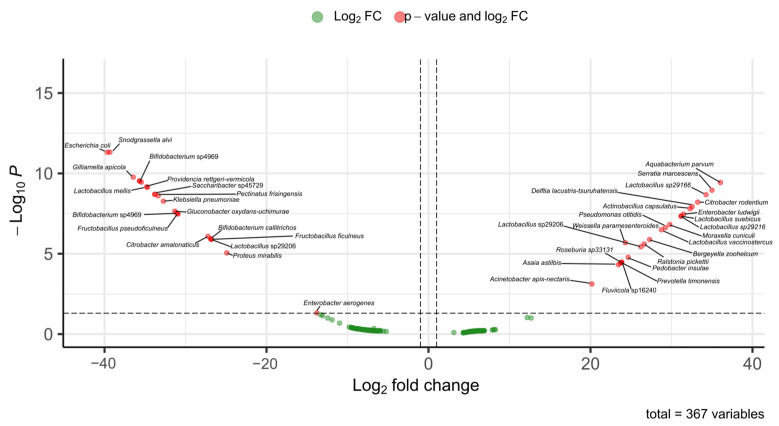
Differentially abundant bacterial taxa between *Melipona* and *Trigona* based on fold-change analysis. The volcano plot shows ASVs with significant differences in abundance between the microbiomes of *Melipona* and *Trigona*. Red dots indicate bacterial taxa with both significant *p*-values and log2 FC, while green dots correspond to taxa with fold change values that did not reach statistical significance. Positive log2 FC values indicated enrichment in *Trigona*, whereas negative values indicated enrichment in *Melipona*. A total of 367 bacterial variables were analyzed.

Among stingless bees, vulture bees are specialized in the consumption of rotten meat. Studies conducted to determine whether the gut microbiota of vulture bees differs from that of bees that feed on nectar and pollen have shown that their microbiome composition is different, likely due to their diet types. It has been suggested that bacteria capable of producing lactic and acetic acids may play a role in digestion and pathogen defense [45]. In our study, two species identified as vulture bees, *Trigona corvina* and *P. bilineata*, were included. These species are facultative necrophages, and the relative abundance of their gut microbiota differs from that of pollinivorous species such as *M. beecheii* (Figure 7).

In previous studies on these species, Maccaro et al. [46] reported the presence of bacteria such as *Commensalibacter*, *Pseudomonas*, *Escherichia*, and *Enterobacter* in facultative necrophagous bees. In our study, we identified some of these bacterial genera in *Trigona* spp. classified as facultative necrophages. Several of the bacteria found in high abundance in these bees are considered opportunistic human pathogens and are known for their antibiotic resistance and ability to form biofilms, including *Pseudomonas*, *Myroides*, *Bacteroides*, *Serratia*, *Enterococcus*, *Klebsiella*, *Salmonella*, and *Enterobacter* [47,48,49]. The ability to form biofilms in response to environmental stress or antimicrobial compounds from competing microbes may confer a symbiotic advantage to bees [46].

In another study, Ntougias et al. [49] found that the obligate necrophagous bee harbored several bacterial taxa associated with fatty or salted meat, including *Carnimonas nigrificans*, *Lactobacillus* sp., *Halomonas* sp., and *Hatalea alkaline*. In our study, we also detected *C. nigrificans* in *T. corvina*. In this context, Maccaro et al. [46] found that several bacteria are likely acquired from the environment but selectively retained based on dietary needs. Similarly, Figueroa et al. [45] suggest that the vulture bee microbiome adapts to the host’s novel diet through a combination of novel symbiont recruitment, the loss of some ancestral microbes, and the possible adaptation of others.

Overall, the observed differences in microbiota composition among species and genera likely reflect a combination of dietary specialization, environmental exposure, and phylogenetic history. However, one limitation of our exploratory study is the absence of independent biological replicates for each species, which restricts our ability to assess species-specific microbiome variability. Although we included 10 individuals per species, all were sampled from a single colony or locality, limiting the scope of ecological and geographic comparisons within species. Future studies, including multiple colonies and environmental contexts, will be essential to validate the observed taxonomic patterns and to further understand the factors shaping gut microbiota diversity in stingless bees.

## 5. Conclusions

This study offers a novel perspective on the diversity and predicted composition of gut microbiota in Mexican stingless bees. By revealing differences in microbial communities among species with different feeding habits, our findings emphasize the importance of preserving microbial biodiversity in these bees. Considering that many stingless bee species are threatened by environmental pressures such as habitat loss and competition from introduced species, conserving their associated microbiota could be key to ensuring their survival.

## Data Availability

The original contributions presented in this study are included in the article/Supplementary Materials. Further inquiries can be directed to the corresponding author.

## Supplementary Materials

The following supporting information can be downloaded at https://www.mdpi.com/article/10.3390/microorganisms13071645/s1. Table S1: Collection dates and localities of stingless bee species (species, locality, coordinates, date, altitude, and identifier). File Genus-Species. File sv. Sequences.
