# Supplementary material for: Gut Microbiota Diversity in 16 Stingless Bee Species (Hymenoptera: Apidae: Meliponini)
Source: Microorganisms. 2025 Jul 11;13(7):1645. doi: 10.3390/microorganisms13071645 (PMC12299229; doi:10.3390/microorganisms13071645)
Supplement: Supplementary file 1 [file microorganisms-13-01645-s001.zip › Table S1 Collection dates.docx]

Table S1 Collection dates, localities of stingless bee species.

| **Species** | **Locality** | **Coordinates** | **Date** | **Altitude** | **Identifier** |
| --- | --- | --- | --- | --- | --- |
| *Cephalotrigona zexmeniae* | Quintana Roo | 20.86789, -88.63165 | 20-IV-2023 | 50 m.a.s.l. | CIBE 23-010 |
| *Frieseomelitta nigra* | Oaxaca | 17.899444, -96.361667 | 08-X-2023 | 1 560 m.a.s.l. | CIBE 23-001 |
| *Melipona beecheii* | Oaxaca | 17.899444, -96.361667 | 08-X-2023 | 1 560 m.a.s.l. | CIBE 19-001 |
| *Melipona fasciata* | Oaxaca | 17.0320856, -97.9293247 | 16-IV-2023 | 1 560 m.a.s.l. | CIBE 23-013 |
| *Melipona solani* | Chiapas | 14.97740, -92.26497 | 16-X-2022 | 650 m.a.s.l. | CIBE 22-012 |
| *Melipona yucatanica* | Quintana Roo | 20.86789, -88.63165 | 20-IV-2023 | 50 m.a.s.l. | CIBE 23-009 |
| *Nannotrigona perilampoides* | Tabasco | 17.84092, -92.61893 | 08-XI-2022 | 20 m.a.s.l. | CIBE 22-015 |
| *Partamona bilineata* | Oaxaca | 17.899444, -96.361667 | 08-I-2023 | 1 560 m.a.s.l. | CIBE 23-002 |
| *Plebeia frontalis* | Quintana Roo | 20.86789, -88.63165 | 20-IV-2023 | 50 m.a.s.l. | CIBE 23-011 |
| *Plebeia llorontei* | Oaxaca | 17.899444, -96.361667 | 30-VII-2023 | 1 560 m.a.s.l. | CIBE 23-020 |
| *Plebeia melanica* | Oaxaca | 17.899444, -96.361667 | 08-V-2021 | 1 560 m.a.s.l. | CIBE 23-021 |
| *Scaptotrigona hellwegeri* | Oaxaca | 17.0320856, -97.9293247 | 16-IV-2023 | 1 560 m.a.s.l. | CIBE 23-014 |
| *Scaptotrigona mexicana* | Chiapas | 14.97740, -92.26497 | 08-I-2023 | 650 m.a.s.l. | CIBE 22-011 |
| *Scaptotrigona pectoralis* | Oaxaca | 17.899444, -96.361667 | 08-V-2021 | 1 560 m.a.s.l. | CIBE 19-002 |
| *Trigona corvina* | Oaxaca | 18.06149, -96.39823 | 30-VII-2023 | 1 560 m.a.s.l. | CIBE 19-006 |
| *Trigona fulviventris* | Tabasco | 17.84092,-92.61893 | 08-XI-2022 | 20 m.a.s.l. | CIBE 22-016 |
